# Supplementary material for: Whole Tumor Histogram Analysis Using DW MRI in Primary Central Nervous System Lymphoma Correlates with Tumor Biomarkers and Outcome
Source: Cancers (Basel). 2019 Oct 8;11(10):1506. doi: 10.3390/cancers11101506 (PMC6827036; doi:10.3390/cancers11101506)
Supplement: Supplementary file 1 [file cancers-11-01506-s001.pdf]

Supplementary Materials

Table S1. Summary of mean values of ADC histogram parameters.

Parameter	Mean	SD	Min	Max
Minimum	477.502748	264.965564	−256	1116
Maximum	1524.4584	656.539304	135	3826
Mean	854.916404	328.481986	74.9096859	1742.13482
Standard Deviation	161.416139	82.2013793	10.1672321	480.820994
ADC_{1}	566.584289	234.248578	47	1205.64
ADC_{5}	630.146579	252.647978	49.8	1351.1
ADC_{15}	696.967043	273.171824	59	1464.1
ADC_{16}	740.20063	288.277534	62	1525
ADC_{75}	949.46049	366.028907	82	1948
ADC_{95}	1149.98729	456.658557	98	2607.4
ADC_{99}	1310.78852	534.709867	109.96	3214.1
ADC Skewness	0.70058876	0.52654495	−1.0129279	2.16832945
ADC Kurtosis	4.09562495	1.50679093	1.85150601	8.73794856
WM_{Min}	575.317177	211.18682	44	1198
WM_{Max}	811.149802	309.310145	83	1968
WM_{Mean}	688.658343	252.255017	70.0784314	1484.54673
WM_{SD}	48.4144682	25.5357461	3.51379334	149.331673
WM_{1}	585.876283	213.223575	45.56	1222.7
WM_{5}	610.73079	222.715548	54	1265.4
WM_{15}	637.639054	232.355639	58.8	1313.8
WM_{25}	654.502431	238.778132	63	1367
WM_{75}	722.207899	266.314159	75	1593
WM_{95}	768.674098	284.533744	79.5	1729.4
WM_{99}	800.017998	301.525502	82.9	1864.56
WM Skewness	0.09640038	0.39675475	−1.0089145	1.17621439
WM Kurtosis	2.85275338	0.68178171	1.81001633	5.60791992
$nADC_{Min}$	0.85564686	0.37916413	−0.3815201	1.84768212
$nADC_{Max}$	1.89212724	0.47470022	0.73548387	3.18963338
$nADC_{Mean}$	1.25451006	0.27695343	0.49461055	2.36769555
$nADC_{1}$	0.98675921	0.28406564	0.20610483	1.97467857
$nADC_{5}$	1.04802127	0.27864378	0.25187707	2.16141417
$nADC_{15}$	1.10773235	0.27516706	0.32084194	2.2606346
$nADC_{75}$	1.32795052	0.29070075	0.60310219	2.3933287
$nADC_{95}$	1.51144321	0.33808471	0.63047656	2.55381181
$nADC_{99}$	1.65392267	0.38383613	0.63411587	2.75167925

SD = standard deviation. ADC = apparent diffusion coefficient. nADC = normalized ADC ratios computed as the ratio of the ADC values within a lesion to the ADC values within normal white matter. WM = white matter.

Table S2. Correlations of non-normalized ADC values with Ki-67 in patients with available data. Results considered significant (*) when $p < 0.05$.

Parameter	All Patients (n = 28)		No Hemorrhage (n = 18)		HIV+ (n = 2)		HIV− (n = 26)	
	r	*p*-value	r	*p*-value	r	*p*-value	r	*p*-value
ADC_{Min}	0.136	0.49	0.578	0.01*	--	--	0.183	0.37
ADC_{Max}	0.004	0.99	0.325	0.19	--	--	0.068	0.74
ADC_{Mean}	0.074	0.71	0.533	0.02*	--	--	0.154	0.45
ADC_{SD}	−0.022	0.91	0.286	0.25	--	--	0.034	0.87
ADC_{1}	0.147	0.46	0.591	0.01*	--	--	0.219	0.28
ADC_{5}	0.117	0.55	0.575	0.01*	--	--	0.194	0.34
ADC_{15}	0.087	0.66	0.549	0.02*	--	--	0.167	0.42
ADC_{16}	0.084	0.67	0.543	0.02*	--	--	0.164	0.42
ADC_{75}	0.063	0.75	0.521	0.03*	--	--	0.143	0.49
ADC_{95}	0.054	0.78	0.493	0.04*	--	--	0.130	0.53
ADC_{99}	0.052	0.79	0.478	0.045*	--	--	0.125	0.54

ADC = apparent diffusion coefficient.
